# A Systematic Review of Gender Dysphoria Measures in Autistic Samples

**DOI:** 10.1007/s10508-024-02896-4

**Published:** 2024-06-03

**Authors:** Karl Mears, Dheeraj Rai, Punit Shah, Kate Cooper, Chris Ashwin

**Affiliations:** 1 Department of Psychology, Centre for Applied Autism Research, University of Bath, Bath, BA2 5LS UK; 2 Centre for Academic Mental Health, Avon and Wiltshire Partnership NHS Mental Health Trust, Bristol Medical School, University of Bristol, Bristol, UK; 3 Department of Clinical, Educational and Health Psychology, University College London, London, UK

**Keywords:** Autism, Gender dysphoria, Gender incongruence, Systematic review

## Abstract

This systematic review investigated how studies have measured gender dysphoria (GD) in autistic samples and the impact of using different measures on study results. The literature search identified 339 relevant papers, with 12 of them meeting the inclusion criteria. Results showed that seven different measures of GD characteristics have been used with autistic samples and that the studies consistently reported a greater number of GD characteristics and a greater severity of GD in autistic compared to non-autistic samples. Methodological common practices were found in recruiting participants from clinical settings rather than the general population, having more autistic males than females in the samples, for studies being conducted in Europe, North America, and Oceania, and using single-item measures of GD for samples of autistic children. Issues were identified with study designs and measures of GD, suggesting a need for a more standardized multi-item self-report measure of GD for use in clinical and non-clinical samples across different ages and cultures.

## Introduction

The present systematic review uses identity-first language (i.e., autistic person) throughout as it is generally the preferred language reported by the autism community (Kenny et al., 2016; Taboas et al., 2023). Higher rates of gender diversity have been reported in autistic people when compared to non-autistic people (e.g., Janssen et al., 2016). Initial findings indicate that gender diversity is higher in autistic people assigned female at birth than autistic people assigned male at birth (e.g., Bejerot & Eriksson, 2014; Corbett et al., 2023). Gender diversity is a term used to describe people whose gender identities and/or expressions of gender are different from social and cultural expectations attributed to their sex assigned at birth. This may include, for example, non-binary and gender non-conforming identities, and there are others who do not identify as cisgender, as well as many other culturally diverse identities (Coleman et al., 2022). Gender diversity does not imply experiencing distress and is not a clinical mental health condition.

A subset of gender-diverse people may experience gender dysphoria (GD) which is a state of distress due to an incongruence between one’s experienced or expressed gender and one’s assigned gender or biological sex at birth, leading to a persistent desire to change one’s gender (APA, 2013). GD has previously been described in the literature in two key ways, with one view approaching GD as a continuous construct with GD symptoms existing in varying degrees within individuals (de Vries & Cohen-Kettenis, 2012; Schneider et al., 2016). An alternative view is to view GD as a categorical construct based on a number of symptoms where an individual either does, or does not, meet diagnostic criteria for GD (APA, 2013). The current diagnostic criteria for GD states adolescents and adults must experience at least two specific aspects of the diagnostic criteria for GD for a minimum of six months (APA, 2013), such as a marked incongruence between one’s experienced or expressed gender and primary and/or secondary sex characteristics, a strong desire for the primary and/or secondary sex characteristics of another gender, and a strong desire to be the opposite sex. The condition must also be associated with clinically significant distress or impairment in social, occupational, or other important areas of functioning. The diagnostic criteria for children are adapted to developmental differences in gender expression before adolescence. For example, to meet diagnostic criteria children must have a strong preference for the clothes typical of another gender, and a strong preference for the toys, games, or activities stereotypically used or engaged in by another gender.

There has been increased interest and research into the relationship between autism and GD, following reports of an overrepresentation of individuals with autism in gender clinics (e.g., de Vries et al., 2010). The majority of the research takes the view of GD being a categorical construct where participants either have or have not a diagnosis of GD (e.g., de Vries et al., 2010), or have scores on a questionnaire for GD that are indicative of a GD diagnosis (e.g., Kallitsounaki & Williams, 2020). Research has reported that up to 26% of people attending gender clinics have an autism diagnosis (Kaltiala-Heino et al., 2015; Morandini et al., 2022; Robinow & Knudson, 2005). These rates are notably higher than the prevalence rates for autism of approximately 1.76% reported within the general population (Roman-Urrestarazu et al., 2021).

Research has also shown that people with GD score highly on autism trait measures (Skagerberg et al., 2015) report having a greater number of autistic traits compared to cisgender non-autistic people (Akgül et al., 2018; van der Miesen et al., 2018a), but do not report greater number of autistic traits when compared to autistic people (van der Miesen et al., 2018a). Warrier et al. (2020) studied a very large sample (*N* = 641,860) consisting of cisgender, transgender and gender-diverse individuals and reported that transgender and gender-diverse individuals had significantly higher rates of autism diagnoses and autistic traits when compared to those who reported being cisgender. These findings provide evidence from a very large sample that higher autistic traits are prevalent in those individuals who are most likely to experience GD. However, it does not inform us about whether autistic samples differ in their degree of GD compared to non-autistic samples. Therefore, evidence for greater GD in people with a diagnosis of autism compared to controls would also provide strong evidence for the association between autism and GD. Three recent reviews of the literature have examined the prevalence of GD and gender diversity in autistic individuals (Kallitsounaki & Williams, 2023; Manjra & Masic, 2021; Thrower et al., 2020). While the reviews included studies which provide compelling evidence toward the link between autism and GD (e.g., George & Stokes, 2018; Kallitsounaki & Williams, 2020), the reviews did not do an in-depth evaluation of the methodologies used within these papers. For example, they did not highlight about the implications the study designs could have on the research results, and Manjra and Masic (2021) focused on child and adolescent samples meaning a comprehensive summary on autistic adults was still missing.

Indeed, there are issues with how GD has been measured in research studies in general. A review of the key measures of GD and transgender-related issues used for research purposes identified twenty different instruments (Shulman et al., 2017). Eight of the measures assessed aspects of adjustment and functioning for transgender and gender non-conforming people within their gender non-conforming communities. While these measures do not encompass all the features associated with the diagnostic criteria for GD, they do focus on the social factors which contribute to the distress associated with the experience of GD (Cooper et al., 2020). The remaining 12 instruments identified in the review encompass a broader scope of clinical features for past and present gender related psychiatric diagnoses. More recently, a review of key measures of gender identity, gender expression, or gender dysphoria in transgender and gender-diverse children and adolescents found 24 measurement tools used for research purposes (Bloom et al., 2021). Of these 24, one measured both gender identity and GD (Gender Identity/Gender Dysphoria Questionnaire for Adolescents and Adults; GIDYQ-AA; Deogracias et al., 2007), and four of these, they argued, exclusively measured GD (Body Image Scale, Lindgren & Pauly, 1975; Body Uneasiness Test, Cuzzolaro et al., 2006; Gender Feeling Amplitude, Riley, 2017; and Utrecht Gender Dysphoria Scale, Steensma et al., 2013). Closer inspection of the measures from these two reviews reveals they are limited in their appropriateness for measuring GD for a variety of reasons, such as using outdated language in the Minnesota Multiphasic Personality Inventory Gender Dysphoria subscale (Althof et al., 1983), or items being worded in such a way that may not necessarily capture the GD experience of non-binary people, like the GIDYQ-AA (Deogracias et al., 2007). The most recent measure to be developed is the Gender Diversity and Autism Questionnaire (Strang et al., 2023), which is a self-report tool for autistic transgender young adults to communicate their experiences and needs pertaining to their gender identity. While the questionnaire as a whole is specific to gender diversity, the subscale “tasks and experiences of everyday life” could be associated with a characteristic of GD because of the DSM-5’s suggestion that GD must be associated with significant impairment in social, occupational, or other important areas of functioning. However, due to how recent the questionnaire has been developed the questionnaire has not yet been used in published research.

One scale which has been used in research, which was not included in the literature reviews mentioned above, is the Child Behavior Checklist (CBCL; Achenbach & Rescorla, 2001). The CBCL is a multi-item caregiver-report measure for behavioral problems and broad psychological functioning. A dataset of clinical and control participants that have completed the CBCL is available for researchers to use as an efficient and convenient data sample. Although the measure was not designed to measure gender diversity or gender dysphoria, Item 110 from of measure asks parents to rate how truthful the following statement is when describing their child: “Wishes to be the opposite sex.” This specific item has been used in studies to measure gender diversity within autistic children and adolescents, to which they have found more endorsement of wishing to be the opposite sex for autistic children and adolescents compared to non-autistic children and adolescents (e.g., Janssen et al., 2016; May et al., 2017). However, Item 110 of the CBCL also captures a key characteristic of GD since it measures one’s wish to be the opposite sex, which is one of the diagnostic criteria for GD. The item has also been used in gender care clinics, with strong positive correlations reported between endorsement of Item 110 of the CBCL and diagnosis of Gender Identity Disorder (GID) (Cohen-Kettenis et al., 2003). It is important to highlight that the single item does not also measure distress in relation to a person’s gender identity, which is another key criteria of GD, so it is limited in its ability to accurately measure the entirety of GD. This item has been utilized within research in the GD literature because there have not been many alternative measures available in the field and because of the availability of existing datasets including that measure which are available for researchers. For this review, a “measurement of GD” was considered as a measure that captures any characteristic of GD and any aspect of the DSM-5 diagnostic criteria, i.e., marked incongruence between one’s experienced or expressed gender and primary and/or secondary sex characteristics, a strong desire for the primary and/or secondary sex characteristics of another gender, and a strong desire to be the opposite sex, and clinically significant distress or impairment in social, occupational, or other important areas of functioning.

Some systematic reviews have identified measures for GD (e.g., Bloom et al., 2021; Shulman et al., 2017), and others have explored the relationship between autism and GD (e.g., Kallitsounaki & Williams, 2023; Thrower et al., 2020). There are currently no reviews which are specifically focused on GD within autism, and which offer a critical summary and evaluation of the overall study designs, as well as the measures for GD that have been used in autistic samples. Considering that issues have been raised above about measures of GD, a review of measures of GD that have been used in autistic samples and the findings from these studies would be important to the field to understand the accuracy of the reported relationship between autism and GD and to improve the methodologies for future research in this area. This is of particular importance as the existing measures for GD have not been validated with autistic populations. The aims of the present systematic review were: (1) to identify which measures of GD have been utilized in autistic samples, (2) to critically evaluate the key characteristics of the measures applied to autistic people, (3) to evaluate the design of the studies investigating the relationship between GD and autism using these measures of GD, and (4) to compare the results when different measures of GD have been used to investigate if research findings have been consistent across the applied measures.

## Method

### Search Strategy

The databases of PsycInfo, PubMed, Embase, Scopus and Web of Science were explored using the terms: (gender dysphori* OR gender identity disorder OR gender incongruen* OR transsexualism OR transgender) AND autis* in the Title and Abstract search fields of the databases. Publications had to be from 1980 due to the first gender-related psychiatric diagnoses being introduced in the DSM-III (APA, 1980). The first literature search was conducted on 3 June 2020 and 289 papers were found. To ensure the collated papers were up to date throughout the publication process, the literature search was repeated on 4 August 2020, 7 July 2022, 22 December 2022, 4 August 2023, and 21 April 2024. An additional total of 49 papers were found across the repeated literature searches. The PRISMA guidelines (Page et al., 2021) were referred to in the development of this systematic review. A protocol for this study was pre-registered on PROSPERO (CRD42020173453).

### Inclusion Criteria

Measuring GD in autism is an under-researched area, and so, the inclusion criteria of the review were developed to be as inclusive as possible to avoid missing any papers (see Table 1). Previous research has been inconsistent with its use of terminology for capturing GD, with terms such as “gender incongruence,” “gender dysphoria,” and “transsexualism” being used for the same phenomena, though this may be due to changes within the diagnostic criteria, such as Gender Identity Disorder in the DSM-IV (APA, 1994) and Transsexualism in the DSM-III (APA, 1980), and societal views at the time of that study. Yet, the wish to be another gender or sex has consistently been a diagnostic criterion for gender related psychiatric diagnoses since 1980. Due to this, a “measurement of gender dysphoria” was operationally defined as a measure that captures any characteristic of GD and any aspect of the DSM-5 diagnostic criteria for the condition.

**Table 1:** Inclusion and exclusion criteria

	Inclusion	Exclusion
Participants	All ages All genders Diagnosis of autism is required for at least one study sample group	
Study design	Quantitative and systematic reviews No minimal sample size A measure for GD must be administered with an autistic sample	Qualitative No focus on Autism No focus on GD No focus on GD and Autism No measure of GD applied to autistic samples
Publication	Peer-reviewed Published in English language Must be published from 1980 due to the first gender-related psychiatric diagnoses being introduced in the DSM-III (3rd ed.; *DSM–III*; APA, 1980)	Grey literature Not in English

*GD* = Gender dysphoria

At least one measurement of GD was needed to have been administered to the participants for the study to be included in this review. Study samples were not required to have a diagnosis of GD or previous gender-related psychiatric conditions, but study samples were required to have at least one group of individuals who had a formal diagnosis of autism. Other sample groups could also be recruited, but, in this case, scores for GD must be compared between the autistic group and any other comparative group during the statistical analysis.

### Screening

**Fig. 1:**
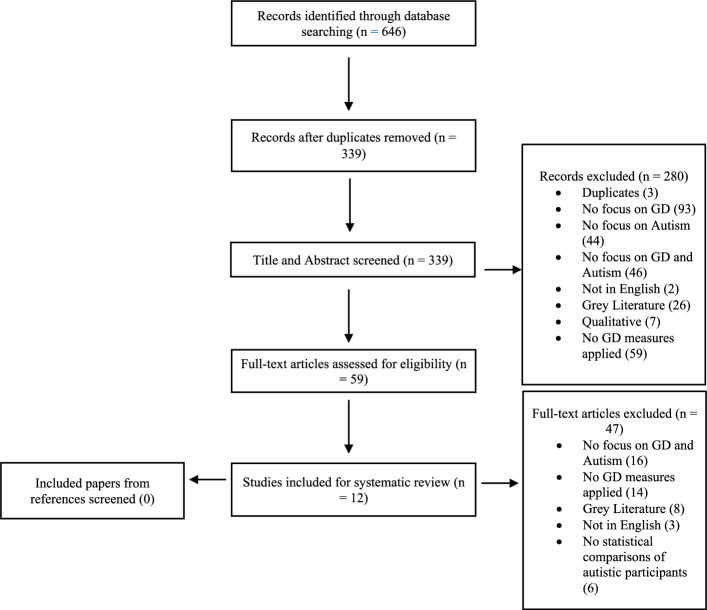
PRISMA chart detailing studies approved per stage of the screening process

Following the recommendations of Pollock and Berge (2018), articles were screened in three stages. Initially, articles were screened by title and abstract for their relevance. Titles and abstracts were deemed irrelevant if autism and GD were not mentioned, if there was no indication of GD being measured, and if the inclusion requirements were not met. The full text of the remaining studies was then reviewed. Reference lists for all full-text articles were then checked to search for additional relevant studies. See Fig. 1 for a detailed overview of the screening process. Twelve studies were included in the final analysis, from an initial 339 papers.

### Inter-rater Reliability

Two authors of this paper (KM and KC) screened studies using the inclusion and exclusion criteria, and both screened 100% of the articles at the Title/Abstract, and Full Text levels. The two reviewers worked independent from each other to avoid a single reviewer introducing a biased interpretation of the review criteria. A third researcher (CA) was consulted if there were any disputes for article screening. Agreement between the two researchers, and the Cohen’s kappa analysis, was measured by the Covidence software for Systematic Reviews. For the Titles and Abstracts, Cohen’s kappa was 0.45, which suggested a fair level of agreement (McHugh, 2012). For the screening of the full texts, Cohen’s kappa was 0.63, which indicated a substantial level of agreement for that particular phase (McHugh, 2012).

### Quality Assessment

The widely used CASP checklist (Critical Appraisal Skills Program, 2018) was used to assess the quality of the studies included in the review and was adapted into 12 questions (see Table 2). Similar to the procedure of Duggleby et al. (2010), a three-point rating system was developed and used to determine scores for each article for each of the CASP’s 12 questions. A weak score (1 point) was assigned to CASP items when the study offered little to no justification or explanation for a particular issue (e.g., where, when, or how the data were collected was not mentioned). A moderate score (2 points) was given to items that addressed the issue but did not fully elaborate on it (e.g., autism diagnoses were screened and verified but the procedure in which this was done by was not explained). A strong score (3 points) was assigned to items that extensively justified and explained the issue at hand (e.g., the data was screened for outliers and tests of normality were completed, or if confounding variables were considered in the analysis). The abbreviation “N/A” was used for question four when no control group was utilized. The scores for all 12 items were totaled and then divided by how many items were appropriate for that study to give the study a mean CASP score, with mean CASP study scores ranging from 1 (a weak study) to 3 (a strong study). All studies were included within the results of this review, irrespective of their quality. Prior to all 12 studies undergoing a quality assessment, three of the 12 papers (25%) were randomly selected for two authors (KM and KC) to complete quality assessments for. Out of a total of 36 items, there was agreement in scores for 29 of these items (80.6%). The remaining nine studies were then assessed by one author (KM).

**Table 2:** CASP quality assessment for included studies

CASP item number	Bedard et al. (2010)	Strang et al. (2014)	Janssen et al. (2016)	May et al. (2017)	Cooper et al. (2018)	George and Stokes (2018)	van der Miesen et al. (2018b)	Kallitsounaki and Williams (2020)	McPhate et al. (2021)	Kallitsounaki and Williams (2023)	Corbett et al. (2023)	Mo et al. (2024)
Did the study address a clearly focused issue?	3	3	3	3	3	3	2	3	3	3	3	3
Did the authors use an appropriate method to answer their question?	3	3	3	3	3	3	2	3	2	3	2	3
Were the cases recruited in an acceptable way?	2	3	3	2	2	2	2	1	3	2	3	3
Were the controls (if applicable) selected in an acceptable way?	N/A	3	2	2	2	2	2	N/A	3	2	3	3
Was an autism diagnosis accurately measured / diagnosed / screened / confirmed?	3	3	3	3	2	2	3	2	3	2	3	3
Were the measures of gender dysphoria of high quality?	2	1	1	1	1	2	1	2	1	2	1	2
Have ethical issues been taken into consideration?	3	2	3	3	3	2	3	3	2	3	3	3
Have the authors taken account of the potential confounding factors in the design and/or in their analysis?	2	2	2	3	1	2	2	1	2	2	2	3
Was the data analysis rigorous?	1	1	2	3	2	3	3	3	2	1	2	2
Do you believe the results?	1	2	2	3	2	3	3	3	2	3	2	3
Can the results be applied to the population of interest?	1	2	2	2	3	2	2	2	2	2	2	2
Do the results of this study fit with other available evidence?	1	3	2	3	3	3	3	3	2	3	2	2
Mean study CASP scores	2.0	2.3	2.3	2.6	2.3	2.7	2.3	2.4	2.3	2.3	2.3	2.7

The CASP scores indicate: 1 = weak quality, 2 = moderate quality, 3 = strong quality

### Data Collection and Items

Data were extracted from each included study and collated onto an electronic spreadsheet. There were four areas of interest for the data extraction: (1) characteristics of the measures for gender dysphoria, i.e., number of items, parent vs self-report; (2) participant characteristics, i.e., age, verification of autism diagnosis, sex assigned at birth; (3) study characteristics, i.e., sample size, country the study took place, and recruitment method (clinical settings vs general population); and (4) main findings of the research. The data were then synthesized using narrative synthesis. Tables were also produced and reported to provide information and comparison of the methodologies and findings of the reported studies.

## Results

### Quality of the Included Studies

The mean CASP scores for each of the studies in this systematic review ranged from 2.0–2.7. All 12 studies were considered to have been of moderate–high quality regarding the appropriateness of the methods used, accuracy of the reported autism diagnoses of the participants, and ethical considerations. The authors felt that the quality of the GD measures used in the 12 studies was of weak–moderate quality. Seven of the 12 studies used measures of low quality, and five of the 12 studies used measures of moderate quality.

### Measures of Gender Dysphoria Applied to Autistic Participants

Seven measures of GD characteristics have been applied to autistic participants in published research studies (see Table 3). Four out of seven measures (57.1%) were single-item measures, and three of the seven measures were multi-item measures (42.9%). Three of the single-item questions were drawn from existing multi-Item measures (CBCL Item 110, Achenbach & Rescorla, 2001; Adult Self-Report Item 110; Achenbach & Rescorla, 2003; Youth Self-Report Item 110, Verhulst et al., 1997), and these selected single-Item questions are grammatical variations of the same question: “Do you wish to be the opposite sex?” One of the multi-item measures is a subscale from a larger multi-item measure which measures body-related GD (Gender Diversity Screening Questionnaire-Informant, GDSQ-I, subscale Body Gender Incongruence, Strang et al., 2017). One of the multi-item measures was designed to measure the DSM-IV diagnostic criteria of Gender Identity Disorder in children (Johnson et al., 2004).

**Table 3:** Measures for gender dysphoria identified that have been applied to autistic samples

Measure	Number of items	Parent / Self-report	Scoring	Study measure was applied in
Child Behaviour Checklist, Item 110 – “Wishes to be of opposite sex?” (CBCL, Achenbach & Rescorla, 2001)	1	Parent-report	Item is rated via three responses: “not true,” “somewhat or sometimes true” and “very true or often true.” Research has organized these responses into two groups: those that wish to be the opposite sex (“sometimes,” “often”), and those that do not (“never”)	Corbett et al. (2023); Janssen et al., 2016); May et al. (2017); McPhate et al. (2021); Strang et al. (2014)
Gender Diversity Screening Questionnaire-Informant, Body Gender Incongruence subscale (GDSQ-I, Strang et al., 2017)	3	Parent-report	Items are rated on a 4-point scale. The response options are: “Never true,” “sometimes true,” “often true,” “always.”	Corbett et al. (2023)
Gender Identity/Dysphoria Questionnaire for Adolescents and Adults (GIDYQ-AA, Deogracias et al., 2007	27	Self-report	Items are rated on a 5-point scale, with the past 12 months as the time frame. The response options are: “Always” (coded as 1), “Often” (2), “Sometimes” (3), “Rarely” (4), or “Never” (5). Mean scores of 3 or below are indicative of GD	Bedard et al. (2010); George and Stokes (2018); Kallitsounaki and Williams (2020; 2023)
Gender Identity Questionnaire for Children (GIQC; Johnson et al., 2004)	16	Parent-report	Items are rated on a 5-point scale for frequency of occurrence (three items also contain a “not applicable” option.) Lower scores reflect greater features of GD in children	Mo et al. (2024)
Original item for “Are you considering, or taking action, to change from your physical sex at birth to your gender identity?” (Cooper et al., 2018)	1	Self-report	Item is rated via two responses: “Yes” or “No.”	Cooper et al. (2018)
Youth Self-Report, Item 110 – “I wish I were of the opposite sex?” (YSR, Verhulst et al., 1997)	1	Self-report	Responses of “never,” “sometimes,” or “often” over the past 6 months. Responses were organized into two groups: those with no wish to be of the opposite gender (“never”) and those who wish to be the opposite gender “sometime” or “often.”	van der Miesen et al. (2018b)
Adult Self-Report, Item 110 – “I wish I were of the opposite sex?” (ASR, Achenbach & Rescorla, 2003)	1	Self-report	Responses of “never,” “sometimes,” or “often” over the past 6 months. Responses were organized into two groups: those with no wish to be of the opposite gender (“never”) and those who wish to be the opposite gender “sometimes” or “often.”	van der Miesen et al. (2018b)

In terms of the type of response for each measure, four out of seven measures (57.1%) were self-report measures, while three measures (42.9%) were parent/informant-response tools. Characteristics of GD were assessed differently across the measures. Four measures (57.1%) consisted of descriptive responses to a single item, whereby responses of “sometimes/sometimes true/always/always true/yes” indicated a characteristic of GD. Two measures (28.6%) produced mean scores, with specific scores indicating the participant may meet clinical criteria for a GD diagnosis (Deogracias et al., 2007; Johnson et al., 2004). One measure (14.3%) produced mean scores, but specific scores did not indicate clinical criteria for a GD diagnosis and instead were scores to be compared between sample groups of interest (Strang et al., 2017).

### Studies Applying Measures of Gender Dysphoria Characteristics to Autistic Samples

Twelve studies published between 2010 and 2024 were identified as applying measures of GD characteristics to autistic samples (see Table 4; Bedard et al., 2010; Cooper et al., 2018; Corbett et al., 2023; George & Stokes, 2018; Janssen et al., 2016; Kallitsounaki & Williams, 2020, 2023; May et al., 2017; McPhate et al., 2021; Mo et al., 2024; Strang et al., 2014; van der Miesen et al., 2018b). All 12 studies reported recruiting participants diagnosed with autism prior to participation, but only six of these papers (Corbett et al., 2023; May et al., 2017; McPhate et al., 2021; Mo et al., 2024; Strang et al., 2014; van der Miesen et al., 2018b) confirmed participants’ diagnosis was achieved or verified through expert clinical impression using the DSM-IV (APA, 1994), DSM-4-TR (APA, 2000) or the DSM-5 (APA, 2013) as well as implementing diagnostic measures such as the Autism Diagnostic Observation Schedule second version (ADOS-2; Lord et al., 2012) or the Childhood Autism Rating Scale (Schopler et al., 1980). The remaining six papers either stated that the source of the diagnosis was not recorded or validated (Cooper et al., 2018) or did not provide any detailed information (Bedard, 2010; George & Stokes, 2018; Janssen, 2016; Kallitsounaki & Williams, 2020, 2023). Therefore, roughly less than half the available studies have verified autism diagnoses.

**Table 4:** Key findings extracted from the included studies

Author (year of publication)	Country of study	Where autistic participants were recruited from	Age group	Sample size	Sex ratio of autistic participants (assigned male: assigned female)	Autism clarification	Main finding in relation to autism and GD
Bedard et al. (2010)	Canada	Clinical – mental health service	Adults (ages 20–64 years)	Total sample of 31 2 autistic 5 schizophrenia 2 down syndrome 3 anxiety disorder 2 syndromes 1 personality disorder 12 controls	1:1	Not reported	The two autistic participants did not meet clinical threshold for GD when measured through the GIDYQ-AA
Strang et al. (2014)	USA	Clinical – clinic for neurodevelopmental disorders	Children and adolescents (ages 6–18 years)	Total sample of 554 165 typically developed controls 147 autistic 126 ADHD 62 epileptic 54 neurofibromatosis	123:24	Diagnosed by a clinician based on the DSM-IV-TR diagnostic criteria	Compared to a control group, the autistic sample were 7.59 times more likely to endorse the wish to be the opposite sex
Janssen et al. (2016)	USA	Clinical – mental health service	Children and adolescents Autistic children and adolescents (mean age = 8.96 years) Control children and adolescents (mean age = 11.74)	Total sample of 2097 492 autistic 1605 controls	409:83	Formal diagnosis by a clinician between 2011 and 2015	Parents of autistic children and adolescents were 7.76 times more likely to endorse their child wished to be the opposite sex than parents of control participants
May et al. (2017)	Australia	Not clear – secondary data from the National Database for Autism Research	Children and adolescents (ages 6–18 years)	Total sample of 3386 176 autistic 1605 referred to mental health services 1605 controls	136:33	Formal diagnosis according to the National Institutes of Health	Significantly more autistic people endorsed the wish to be the opposite sex compared to controls (*p* < .001). No significant difference in endorsement of the wish to be the opposite sex between the autistic group and those referred to mental health services (*p* = .963)
Cooper et al. (2018)	England	General population – social media and local organizations	Adults Autistic males (mean age = 33.2 years) Autistic females (mean age = 30.38 years) Control males (mean age = 32.02 years) Control females (mean age = 35.88 years)	Total sample of 486 219 autistic 267 controls	118:101	Self-report	Autistic participants were significantly more likely to have had or be planning a gender transition than control participants (*p* < .001)
George and Stokes (2018)	Australia	Not clear – national and international autism organizations	Adults Autistic adults (mean age = 31.01 years) Control adults (mean age = 30.2 years)	Total sample of 571 310 autistic 261 controls	90:219:1 (intersex)	Not recorded	Autistic participants reported significantly greater feelings of GD compared to controls (*p* < .001) based on the total scores for the GIDYQ-AA. Autistic participants also reported significantly greater feelings of GD compared to controls (*p* < .001) for all subscales of the GIDYQ-AA
van der Miesen et al. (2018b)	Netherlands	Clinical – autism specialist clinics	Adolescents and adults Autistic adolescents (mean age = 15.98 years) Control adolescents (ages 11–18 years) Autistic adults (mean age = 32.14 years) Control adults (mean age = 29.9 years)	Total sample of 3242 573 autistic adolescents 1016 control adolescents 807 autistic adults 846 control adults	Autistic adolescents, 469:104 Autistic adults, 616:191	Diagnosed according to the DSM-IV diagnostic criteria by a clinician at a mental health clinic	Autistic adolescents were 2.12 times more likely to endorse the wish to be the opposite sex compared to controls. Autistic adolescents assigned male at birth were 2.73 times more likely to endorse the wish to be the opposite sex compared to controls assigned male at birth. Autistic adolescents assigned female at birth were 2.96 times more likely to endorse the wish to be the opposite sex Autistic adults were 2.46 times more likely to endorse the wish to be the opposite sex than control adults. Autistic adults assigned male at birth were 3.76 times more likely to endorse the wish to be the opposite sex. Autistic adults assigned female at birth were 3.02 times more likely to endorse the wish to be the opposite sex
Kallitsounaki and Williams (2020)	England	General population – Amazon’s online crowdsourcing platform MTurk	Adults (mean age = 36.93 years)	Total sample of 101 13 autistic 88 controls	Not recorded	Self-report	Autistic participants were 50.17 times more likely to report clinically significant levels of GD compared to non-autistic participants
McPhate et al. (2021)	Australia	General population – local organizations Clinical – outpatient psychiatric clinic	Children and adolescents (aged 6–18 years)	Total sample of 4944 232 autistic 86 intellectual disability 672 ADHD 64 anxiety 423 depression 76 ODD or conduct disorder 1605 referred to mental health services 1786 controls	Not recorded	Interviewed for clinical diagnoses by a clinician	Significantly more parents of autistic children endorsed their child wished to be the opposite sex compared to the controls of the CBCL dataset (*p* < .001). No other significant differences were found when comparing the autistic group to the other groups
Kallitsounaki and Williams (2023)	England	General population – social media and local organizations	Adults (mean age = 31.01 years)	Total sample of 347 163 autistic 184 controls	79:84	Not recorded	Autistic people reported significantly more gender dysphoric feelings than non-autistic Individuals (*p* < .001)
Corbett et al. (2023)	USA	General population – local organizations and the community Clinical – autism diagnostic clinic	Children Autistic children (mean age = 11.42 years) Non-autistic children (mean age = 11.71 years)	Total sample of 244 140 autistic 104 controls	104:36	Established by a psychologist, psychiatrist, or behavioral pediatrician based on DSM-5 diagnostic criteria	Parents of autistic children scored marginally significantly higher scores for the GDSQ-I body gender incongruence subscale compared to parents of controls (*p* = .054). There was no significant difference in endorsement of their child wishing to be the opposite sex between autistic and control parents (*p* = .260)
Mo et al. (2024)	Canada	Clinical—hospitals for children General population—Ontario universities, and community organizations	Children (mean age = 8.79 years) Autistic children (mean age = 8.69 years) ADHD children (mean age = 8.49) Autism and ADHD children (mean age = 9.00 years) Neurotypicals (mean age = 9.36 years)	Total sample of 291 104 autistic 104 ADHD 17 autistic and ADHD 66 neurotypicals	77:27	Confirmed by a clinician based on DSM-5 criteria or equivalent	Diagnosis of autism was not significantly correlated with mean GIQC score (*p* = .998)

### Age Groups for Studies

Within these studies, the ages of children ranged from 3 to 12 years old, the age of adolescents ranged from 11 to 18 years old. Corbett et al. (2023) however recruited people aged between 10 and 13 years old and refer to these participants as children (mean age for autistic children = 11.42, SD = 1.03; mean age for typically developed children = 11.71, SD = 1.21). For this review, Corbett et al. (2023) will be referred to as recruiting multiple age groups to be consistent with the other included papers. The age ranges for autistic adults were missing for most of the studies, but adults were defined in all studies as being aged 18 years or older. Six out of the 12 studies (50%) recruited multiple age groups within their samples, and six out of the 12 studies (50%) investigated only one age group. Of the six multi-age group studies, five studies (83.3%) recruited both autistic children and adolescents (Corbett et al., 2023; Janssen et al., 2016; May et al., 2017; McPhate et al., 2021; Strang et al., 2014) and one study (16.7%) recruited autistic adolescent and autistic adult participants (van der Miesen et al., 2018b). Of the single-age group studies, no studies recruited autistic adolescents. One study recruited autistic children (Mo et al., 2024), and five studies recruited autistic adults (Bedard et al., 2010; Cooper et al., 2018; George & Stokes, 2018; Kallitsounaki & Williams, 2020, 2023). Collectively, there does not appear to be a common practice in the ages of the participants recruited.

The study which only recruited autistic children (Mo et al., 2024) utilized the Gender Identity Questionnaire for Children (Johnson et al., 2004), which is a multi-itemed measure of GD characteristics specific to children. This was the only study to utilize this measure. Of the five single-age group studies which recruited autistic adults, four studies (80%) used a multi-itemed measure of GD (Bedard et al., 2010; George & Stokes, 2018; Kallitsounaki & Williams, 2020, 2023). All four of these studies utilized the GIDYQ-AA (Deogracias et al., 2007) as the multi-itemed measure. One out of five adult single-age group studies (20%) measured a characteristic of GD using a single item (Cooper et al., 2018).

Of the six studies which recruited multiple age groups within their samples, all six studies (100%) measured characteristics of GD using single items. Five studies used Item 110 of the CBCL for autistic children and adolescents (Corbett et al., 2023; Janssen et al., 2016; May et al., 2017; McPhate et al., 2021; Strang et al., 2014). One study used Item 110 of the YSR with autistic children and Item 110 of the ASR with autistic adults (van der Miesen et al., 2018b). Corbett et al. (2023) also used a multi-measure in addition to the single-item measure, using the GDSQ-I body gender incongruence subscale. Together, these results suggest single-item measures have been utilized to measure characteristics of GD with autistic children and adolescents (for various reasons highlighted above), but it has been common practice to use multi-item measures for GD with adult autistic samples.

Of the five studies which recruited both autistic children and autistic adolescents, all five of these studies relied on parental report (Corbett et al., 2023; Janssen et al., 2016; May et al., 2017; McPhate et al., 2021; Strang et al., 2014). The remaining five studies which recruited autistic adolescents and/or autistic adults relied on self-report responses. This suggests it is common practice in the studies included in this systematic review to use parental report for measuring characteristics of GD when both autistic children and adolescents have been recruited. Conversely, it is common practice to use self-report for measuring characteristics of GD when a study has recruited autistic adolescents and / or autistic adults.

### Proportion of Autistic: Non-Autistic Participants

Overall, there was a higher prevalence of non-autistic control participants recruited within the 12 studies identified. Nine studies (75%) recruited more non-autistic participants than autistic participants (Bedard et al., 2010; Cooper et al., 2018; Janssen et al., 2016; Kallitsounaki & Williams, 2020, 2023; May et al., 2017; McPhate et al., 2021; Strang et al., 2014; van der Miesen et al., 2018b). The remaining three papers (25%) recruited more autistic participants than non-autistic participants (Corbett et al., 2023; George & Stokes, 2018; Mo et al., 2024). The results highlight that no studies recruited the same number of autistic participants as control participants. Despite the differences in sample sizes between comparative groups, no papers implemented power calculations or adjusted the sample sizes to improve on the unequal total sample numbers within groups. Adjustments were only implemented to balance the sex ratios between diagnostic groups by randomly removing participants from one diagnostic group until the sex ratios were equal in two studies (Strang et al., 2014; van der Miesen et al., 2018b).

Seven of the 12 studies (58.3%) recruited and statistically compared autistic and non-autistic samples (Cooper et al., 2018; Corbett et al., 2023; George & Stokes, 2018; Janssen et al., 2016; Kallitsounaki & Williams, 2020, 2023; van der Miesen et al., 2018b). Three of the 12 studies (25%) recruited and statistically compared samples of autistic, controls, and other conditions such as Attention Deficit Hyperactivity Disorder, Depression, and epilepsy (May et al., 2017; McPhate et al., 2021; Strang et al., 2014). One study (8.3%) recruited samples of autistic, ADHD and neurotypicals, but did not make statistical comparisons between these groups (Mo et al., 2024). One study (8.3%) did not recruit a control group and only recruited and statistically compared autistic participants to other developmental disabilities and mental health conditions, such as Down’s Syndrome and Schizophrenia (Bedard et al., 2010).

### Proportion of Male: Female Participants

Nine of the 12 studies (75%) recruited a higher proportion of autistic males in comparison to autistic females (Cooper et al., 2018; Corbett et al., 2023; Janssen et al., 2016; Kallitsounaki & Williams, 2020; May et al., 2017; McPhate et al., 2021; Mo et al., 2024; Strang et al., 2014; van der Miesen et al., 2018b). One study (8.3%) recruited more autistic females than autistic males (George & Stokes, 2018). Two studies (16.7%) recruited similar numbers of autistic males and autistic females (Bedard et al., 2010; Kallitsounaki & Williams, 2023). This highlights a greater number of autistic males have been recruited compared to autistic females when researching characteristics of GD.

### Clinical versus General Population Sample

Four of the studies identified for this review (33.3%) recruited autistic participants from clinical settings. Within these studies, participants were sought from mental health services (Bedard et al., 2010; Janssen et al., 2016), autism specialist clinics (van der Miesen et al., 2018b), and clinics for neurodevelopmental disorders (Strang et al., 2014). Three studies (25%) recruited participants from the general population, using social media and local organizations to recruit autistic participants (Cooper et al., 2018; Kallitsounaki & Williams, 2020, 2023). Three papers (25%) recruited autistic participants from both the general population and from clinical settings. One paper recruited from an outpatient psychiatric clinic (McPhate et al., 2021), another paper recruited from a diagnostic clinic (Corbett et al., 2023), and another recruited from clinical services such as pediatrics and child psychiatry (Mo et al., 2024). It is not clear if the remaining two studies recruited clinical samples or from the general population because George and Stokes (2018) report contacting national and international autism organizations, with no further details, and May et al. (2017) used secondary data from the National Database for Autism Research; therefore, it is not clear if clinical organizations were contacted. These results identify a variety within the included studies of where participants are recruited from. There does not appear to be an established common practice for participant recruitment when investigating characteristics of GD in autism.

### Study Location

The 12 studies have been conducted across five countries. One of these studies (8.3%) was conducted in the Netherlands (van der Miesen et al., 2018b), three studies (25%) were carried out in USA (Corbett et al., 2023; Janssen et al., 2016; Strang et al., 2014;), three studies (25%) were carried out in Australia (George & Stokes, 2018; May et al., 2017; McPhate et al., 2021), three studies (25%) were conducted in England (Cooper et al., 2018; Kallitsounaki & Williams, 2020, 2023), and two studies (16.7%) were conducted in Canada (Bedard et al., 2010; Mo et al., 2024). These results show that the research in this area has been conducted within only the Western world and seems evenly distributed between North America, northern Europe, and Australia.

### Year of Publication

While the search included papers published from 1980, all papers relevant to this review were found from 2010 onwards, with seven of the 12 papers being published between 2010 and 2020, and five of the 12 papers being published from 2020.

### Gender Dysphoria and Autism: Summary of Outcome Measures

#### Presence of Gender Dysphoria Characteristics in Autism

In eight of the 12 studies (66.7%), autistic samples reported greater GD compared to non-autistic samples. Of these eight studies which reported this relationship, five (62.5%) measured GD characteristics using single items (Cooper et al., 2018; Janssen et al., 2016; May et al., 2017; Strang et al., 2014; van der Miesen et al., 2018b), and three of the studies (37.5%) employed multi-item measures (George & Stokes, 2018; Kallitsounaki & Williams, 2020, 2023). In contrast, four out of the 12 studies (33.3%) did not report a relationship between autism and GD characteristics (Bedard et al., 2010; Corbett et al., 2023; McPhate et al., 2021; Mo et al., 2024). Two of these four papers had measured GD using the CBCL item 110 single Item (Corbett et al., 2023; McPhate et al., 2021) whereas Bedard et al. (2010) and Mo et al. (2024) had administered different multi-item measures. One paper reported finding both significant and non-significant differences in GD characteristics between autistic and non-autistic samples (Corbett et al., 2023). A significant difference was found in scores for the multi-item measure (the GDSQ-I) but no significant differences were found on the CBCL Item 110.

Ten of the 12 studies compared autistic people’s responses to measures of GD characteristics to a control group. Of these 10 studies, five found significantly more autistic people endorsed the wish to be the opposite sex than controls (Cooper et al., 2018; Janssen et al., 2016; May et al., 2017; Strang et al., 2014; van der Miesen et al., 2018b) and four papers found autistic people had significantly greater GD on multi-item measures of GD than controls (George & Stokes, 2018; Kallitsounaki & Williams, 2020, 2023; Corbett et al., 2023). Two studies reported mixed results. McPhate et al. (2021) found significantly more autistic people endorsed wishing to be the opposite sex when compared to a secondary dataset comprised of control participants, but there was no significant difference when the autistic group were compared to a locally recruited control group. Corbett et al. (2023) found no significant differences in parental endorsement of their child wishing to be the opposite sex when comparing an autistic group to a control group, but did find parents of autistic children and adolescents endorsed greater body-related GD than parents of non-autistic children and adolescents.

Three of the 12 papers (Bedard et al., 2010; May et al., 2017; McPhate et al., 2021) compared autistic people’s responses to measures of GD characteristics to that of other conditions, and all three found no significant differences when comparing the autistic groups to the other conditions. Interestingly, when papers compared other diagnostic groups responses to characteristics of GD to that of control groups, these diagnostic groups also had significant differences with control groups (McPhate et al., 2021; Strang et al., 2014). One paper (Mo et al., 2024) investigated if autistic and ADHD diagnoses were correlated with characteristics of GD in children, and found neither diagnostic groups were correlated with child-focused GD characteristics.

The greater presence of GD characteristics in autistic compared to non-autistic groups was a consistent finding regardless of whether measures were completed as self-report or parent-report. Yet, there were differences in the degree of “wishing to be the opposite sex” when completed by parents compared to the self. Using parent-reports of Item 110 of the CBCL, Janssen et al. (2016) and Strang et al. (2014) found children and adolescents diagnosed with autism were 7.59–7.76 times more likely to wish to be the opposite sex than a control group with no diagnoses. However, when using self-report for Item 110 of the YSR and ASR, autistic adolescents and adults were 2.12–2.46 times more likely to wish to be the opposite sex compared to non-autistic adolescents and adults (van der Miesen et al., 2018b).

The elevated presence of GD characteristics among autistic participants was a consistent finding across four of the five countries in which the research took place (80.0%). Of the research which did not find a link between autism and GD characteristics, two of the three studies took place in Australia and one study was conducted in North America.

### Sex Differences for Characteristics of Gender Dysphoria in Autistic People

Five out of seven papers (71.4%) which utilized single-item measures explored sex differences. Some research found no sex differences in the wish to be the opposite sex between child, adolescent and adult autistic participants (May et al., 2017) and when compared to control groups (May et al., 2017; Strang et al., 2014;). In contrast, other papers found significantly more assigned female autistic participants expressed a wish to be the opposite sex (van der Miesen et al., 2018b), or planned, or were going to be planning gender transition (Cooper et al., 2018), than male-assigned autistic participants. McPhate et al. (2021) also found a higher proportion of autistic females had been endorsed for wishing to be the opposite sex by their parents compared to autistic males, though this result was achieved through visual inspection of graphical data and not through a formal statistical analysis due to low participant numbers in each group.

Three out of six studies (50%) which utilized multi-item measures explored sex differences. Corbett et al. (2023) found parents of autistic children and adolescents assigned female at birth reported more body-related GD than autistics assigned male at birth. George and Stokes (2018) reported no sex differences were found in total scores for the GIDYQ-AA. However, on individual GD subscales, autistic assigned females scored significantly higher on the “subjective indicators of gender dysphoria” subscale compared to autistic males (*p* < 0.05). Sex differences were also found when GD scores were compared to scores of autistic traits. The relationship between total AQ scores and scores for GD were significant in both assigned males and assigned females (*p* < 0.001), with the relationship being stronger among females than males (*p* < 0.05, George & Stokes, 2018). Kallitsounaki and Williams (2023) also found assigned females reported significantly more gender dysphoric feelings than birth-assigned males through the GIDYQ-AA, but the groups for this analysis included autistic and non-autistic participants and so the authors did not investigate if there were sex differences in GD scores between autistic assigned males and females. The evidence suggests there may be sex differences in reported characteristics of GD, with autistic females reporting more feelings of GD than autistic males. However, most of this evidence comes from the use of single-item measures rather than multi-item measures and so caution should be taken about making inferences from those results.

## Discussion

The results of the systematic review identified seven separate measurement tools within the literature that have been used to measure characteristics of GD in autistic samples, which have been reported in 12 published studies. These 12 studies were of moderate quality, with some key features, such as the measures used, being low quality. A key finding was that autistic participants consistently reported more characteristics of GD compared to controls across all age groups and using all measures. Most of the measures used in the studies included were self-report format and single-item scales. The studies reporting GD in autism have recruited samples from North America, Europe, and Oceania and include more autistic males assigned at birth participants than autistic females assigned at birth. Most studies also recruited participants only from clinical settings. Common practice was identified toward using multi-item measures with adult samples, and studies with child and adolescent samples using single-item measures. Together, the findings here demonstrate more multi-cultural population-based studies are required which administer multi-item scales suitable for measuring GD in autistic samples.

This systematic review found single-item and self-report measures have been the most popular methods for measuring GD within autistic samples. In general, single-item measures should be used with caution, as they often have weaker predictive validity compared to multi-item scales (Diamantopoulos et al., 2012; Sarstedt & Wilczynski, 2009). Examination of the single-item measures in the present study revealed they only addressed the single diagnostic criteria of “a strong desire to be the opposite sex,” while missing out other key diagnostic criteria for GD. Importantly, the single items used in these studies did not measure the distress evoked by the incongruence between a participant’s birth-assigned sex/gender to their current gender identity. The focus on distress regarding one’s assigned gender is a core component to the diagnosis of GD and is very relevant to research with autistic samples as the distress is a key factor reported by people with autism about their GD (Cooper et al., 2021). For these reasons, the authors felt the single-item measures for GD were of low quality in the quality assessment. Therefore, the research on GD in autism using single-item scales reviewed here provide insight into the relationship between autism and characteristics of GD, but may not be aligned with all the diagnostic criteria for GD within the DSM-5. This is turn limits our current understanding of an already under-researched topic.

In contrast, one multi-item measure used to index GD (i.e., the GIDYQ-AA) measures the wider criteria included in the DSM-5 to diagnose GD in adolescents and adults, including experiences of distress. Therefore, multi-item scales used to measure GD may provide an index of GD that is more aligned with the clinical criteria compared to single-item measures. Nevertheless, mixed findings and no group differences in characteristics of GD were found between autistic and control samples when single-item measures were implemented (e.g., Corbett et al., 2023; McPhate et al., 2021) and when using multi-item measures (e.g., Bedard et al., 2010). Elevated GD characteristics were also not correlated with an autism diagnosis in children, when using a multi-itemed measure (e.g., Mo et al., 2024). While the studies by Corbett et al. (2023), McPhate et al. (2021), and Mo et al. (2024) recruited large sample sizes, the study by Bedard et al. (2010) only recruited two autistic people and so conclusions cannot be drawn from the comparisons between the groups from that study. If multi-item measures of GD better represent the full diagnostic criteria, further research employing multi-item measures is needed across all age groups and in larger autistic samples to test this idea.

The present findings also revealed a preference for measures of GD with autistic samples to involve a self-report design. Previous studies have reported significant discrepancies between parent and self-report responses, with self-report responses given by participants found to be more accurate than parental responses (e.g., Ersig et al., 2013; Krain & Kendall, 2000). Since the majority of studies included in the present systematic review recruited autistic children and/or autistic adolescents and relied on parental responses, this raises a question about whether the higher presence of GD consistently found across these age groups is as strong as previously reported, given the known discrepancies between parental and self-report response accuracy.

The results of the present study also found all research measuring GD in autistic participants being carried out in the Western countries, i.e., Netherlands, USA, England, Australia, and Canada. Within Asia, there are fewer people diagnosed with autism or GD compared to Western countries due to differences in the diagnostic procedures and how they are generally utilized in Asia compared to Western countries (Okabe et al., 2008; Qui et al., 2020). Very little is also known about GD in general in other nations, such as African, Middle Eastern, and South American countries, due to the lack of research conducted there on this topic. This means it is currently unknown if the six measures of GD identified within the present study would provide valid measurements of GD for autistic participants outside of Western countries, nor if they are appropriate across different cultures. Future research should develop and translate a standard GD measure into different languages, and recruit participants from wider countries and cultures, to compare the reliability of the existing measures and to test for differences in autistic versus control samples more globally.

The results of this review revealed selection biases in the samples, consistent with previous systematic reviews (e.g., Glidden et al., 2016). Most of the research studies recruited participants from clinical-specific settings, had no conditions other than autism, and recruited more autistic males assigned at birth than autistic females assigned at birth. In addition, half of the studies included in this review did not verify participants’ self-reported claims of having an autism diagnosis. This means the samples of the studies may be unreliable and may not be representative of the wider autistic spectrum. This would question whether the link between autism and GD is evident across the entire autism spectrum. It is also important for future studies to compare GD scores for autistic samples with those reported in other clinical samples, as previous research has shown GD to be associated with other clinical conditions including ADHD (e.g., Thrower et al., 2020), and eating disorders (e.g., Milano et al., 2020). There is also a dearth of research measuring GD in other clinical conditions including depression, anxiety, and psychosis, and comparisons between GD scores in autistic samples and other clinical conditions with non-clinical samples would help to test if feelings of GD might be a more general clinical phenomenon relevant to a large range of conditions.

The results of this review also found no clear bias in recruiting specific age groups. This contrasts with the findings of a previous systematic review (e.g., Glidden et al., 2016), which found most studies exploring the relationship between GD and autism recruited child and adolescent participants. However, there were differences in the aims and the inclusion criteria between the present and former study. For example, the review by Glidden et al. (2016) included all research which was concerned with autism and GD. In contrast, the present study specifically included only studies which applied measures of GD to participants with a diagnosis of autism. Therefore, case studies and quantitative research on autistic children which may have been identified in an earlier review were likely excluded from the present study.

While most studies recruited more birth-assigned autistic males than autistic females, this is to be expected due to the general higher prevalence of boys diagnosed with autism (Loomes et al., 2017). Despite the disproportionate sex ratios in the participants, half of the eleven studies investigated sex differences. Exploration of sex differences was more common in papers that used single-item measures than those that used a multi-item measure. The results of sex differences within and between groups for GD were mixed. Some papers reported no sex differences between autistic participants and control groups, which has been found across child, adolescent and adult samples (May 2017; Strang, 2014). In contrast, other studies did find sex differences between autistic people assigned male or female such that characteristics of GD were elevated in autistics assigned female at birth (Cooper et al., 2018; Corbett et al., 2023; van der Miesen et al., 2018b). Interestingly, Cooper et al. (2018) was the only study to recruit an equal proportion of autistics assigned as either male or female at birth. These male-biased ratios prevent a good understanding of the sex differences in the prevalence and relationship of GD in autism. Although some researchers randomly removed autistic participants to balance the sex ratios (e.g., May et al., 2017; Strang et al., 2014;), this procedure then reduced the power of the statistical analyses and subsequently reduced the reliability of their findings about sex differences. Some autistic people experience gender differently to how non-autistic people experience gender. Autistic people have lower social identification with a gender group than typically developed people, particularly those assigned female at birth, while also experiencing more negative feelings toward a gender group (Cooper et al., 2018). Considering attempts were made in some papers to balance sex ratios, and how few papers used multi-item measures to explore sex differences, conclusions drawn about sex differences should be made with caution. It is important for future research to recruit balanced proportions of autistic males and females and to employ reliable measures in order to better understand the relationship between autism and GD, especially as our current understanding has been impacted by the use of mainly single-item and parent-report measures.

It is important to note that, although the majority of the studies included here found autistic people reported greater GD when compared to non-autistic people, the papers included only captured participants’ levels of GD at a single point of time. This is an important limitation of the included studies as previous research has shown symptoms of GD lessen in some autistic people one year after the initial assessment for GD and before interventions have commenced (e.g., de Vries et al., 2010). That is, feelings of GD may be transient within gender-diverse individuals. While the focus of this review did not explore the duration of GD within autistic samples, it is important to highlight that the included studies considered GD as a series of symptoms, rather than an arbitrary experience for gender-diverse individuals.

Furthermore, as argued previously, single-item measures (e.g., Item 110 of the CBCL) are not wholly representative of the DSM-5’s diagnostic criteria for GD, and instead measure specific characteristics of GD. In contrast, the GIQC and the GIDYQ-AA encompass a greater range of characteristics of GD in children, and adolescents and adults, respectively. Yet, both these measures were developed during the time of the DSM-4 criteria and so they may better reflect Gender Identity Disorder and not simply measure GD criteria per se. Since the research exploring the link between autism and GD has utilized these measures, further research is needed using measures well-aligned with the diagnostic criteria to provide high-quality evidence testing the association between autism and GD. However, there are currently few measures of GD available in the field which are suitably worded, that do not have a binary perspective of gender and are designed to measure GD according to the current diagnostic criteria, and there are even fewer measures that have been designed and validated for use with autistic samples. This situation creates even further discrepancy in the reliability of the conclusions drawn about the link between autism and GD found within the papers included in this systematic review. This suggests there is a need for a new multi-item self-report measure of GD that is suitable for clinical and non-clinical research groups across multiple cultures, that also adheres more closely to the full current diagnostic criteria for GD. This will ensure future research in this area is adequately testing the link between autism and GD, and not autism and gender variance or other factors.

### Conclusion

In conclusion, the present systematic review revealed six different measures of GD which have been used across eleven studies with autistic samples. The research using these measures has consistently reported a higher presence of GD characteristics in autistic samples compared to non-autistic samples, which was evident regardless of the age groups studied. It was also common practice to implement a parent-report single-item measure. Most of the studies recruited more autistics assigned male at birth than assigned female at birth and recruited samples from clinic-specific settings. All studies included in this review were carried out in Europe, North America, or Oceania and included samples recruited from only these regions, demonstrating a gap in studies conducted in other regions such as Asia, Africa, and the Middle East. Given the use of single-item measures, biases in samples and study locations, the strength of the link between GD and autism may not be as strong as the research suggests. Thus, further research is needed to investigate the link between autism and GD by using more reliable multi-item measures of GD with greater diversity within large autistic samples.

## Data Availability

Document of data collation and emerging themes is available upon request.
